# Nasal Carriage of Methicillin-Resistant *Staphylococcus aureus* among Pediatricians in Taiwan

**DOI:** 10.1371/journal.pone.0082472

**Published:** 2013-11-26

**Authors:** Yhu-Chering Huang, Lin-Hui Su, Tzou-Yien Lin

**Affiliations:** 1 Division of Pediatric Infectious Diseases, Department of Pediatrics, Chang Gung Memorial Hospital at Linkou, Kweishan, Taoyuan, Taiwan; 2 Chang Gung University College of Medicine, Kweishan, Taoyuan, Taiwan; 3 Department of Laboratory Medicine, Chang Gung Memorial Hospital at Linkou, Kweishan, Taoyuan, Taiwan; Rockefeller University, United States of America

## Abstract

**Background:**

Health care workers (HCWs) are at the interface between hospitals and communities. The survey for methicillin-resistant *Staphylococcus aureus* (MRSA) carriage among HCWs has mostly been conducted to investigate outbreaks or endemics. Community-associated MRSA are prevalent among children in Taiwan. We conducted this study to better understand the carriage rate of MRSA among pediatricians in non-outbreak situations in Taiwan,.

**Methods:**

A total of 220 pediatricians from Taiwan who attended the annual meeting of Taiwan Pediatric Association in April, 2010 were recruited to participate in this study and were sampled from the nares for the detection of MRSA by polymerase chain reaction (PCR) and further by culture. The following molecular analyses were performed, including pulsed-field gel electrophoresis (PFGE), multilocus sequence typing (MLST), typing of staphylococcal cassette chromosome *mec* (SCC*mec*) and the presence of Panton-Valentine leukocidin (PVL) genes.

**Results:**

MRSA was detected from 15 attendees (6.8%) by PCR. MRSA-colonized attendees had a significantly lower rate (0.041) of working in the medical center, while borderline significantly higher rate of working in the Regional Hospital (p=0.056), than those without MRSA colonization. From those 15 samples, 12 MRSA isolates were identified by culture and molecularly characterized. Three PFGE patterns, two sequence types (ST 59, ST 508), and two SCC*mec* types (IV and V_T_) were identified, respectively. Five isolates, including three carrying SCC*mec* types V_T_, were PVL-positive. All 12 isolates were susceptible to vancomycin, teicoplanin, linezolid, fusidic acid, trimethoprim/sulfamethoxazole, and doxycyclin, and resistant to penicillin.

**Conclusion/significance:**

Around seven percent of pediatricians in Taiwan harbored CA-MRSA in their nares.

## Introduction

Methicillin-resistant *Staphylococcus aureus* (MRSA) is a concern for most hospitals worldwide due to its increasing prevalence [1,2]. MRSA is usually considered a hospital pathogen, but increasingly, it is acquired in the community [3,4]. MRSA infections, though acquired in the community, were traditionally confined to individuals with health care-associated risk factors such as residence in long term care facility, recent hospitalization or surgery, indwelling catheter or hemodialysis [5]. However, the changing epidemiology of MRSA became evident in the 1990s when MRSA infections occurred in previously healthy children without established risk factors for MRSA acquisition [3-5], namely community-associated MRSA (CA-MRSA). CA-MRSA has started to spread from the community into hospitals, where outbreaks have occurred [6,7]. CA-MRSA strains have been recognized as a pathogen which is often genetically different from the healthcare-associated (HA) MRSA, such as relatively limited antibiotic resistance, mostly possessing Panton-Valentine leukocidin genes, usually carrying type IV or V staphylococcal cassette chromosome etc. [3,4].

In Taiwan, CA-MRSA infections have been increasingly reported in pediatric patients since first reported in 2002. MRSA accounting for CA *S. aureus* infections in children without risk factors increased from 9.8% in 1997-2000 to 56% during 2004-05 [8]. Likewise, the nasal MRSA carriage rate for well-child healthcare visits and/or in school children increased from 1.9% in 2001 to ~10.2 % in 2007-2008 [8]. CA-MRSA infections were relatively uncommonly reported in adults in Taiwan and the carriage rate among adults was also relatively low, around 3.8% [8]. Up to 2008, most CA-MRSA isolates in Taiwan shared common molecular characteristics and >80% of the isolates belonged to the clonal complex 59 (sequence type 59 and its variants) [8].

Health care workers (HCWs) are at the interface between hospitals and communities. The survey for MRSA carriage among HCWs has mostly been conducted to investigate outbreaks or endemics but not in non-outbreak situations [9]. Since a substantial proportion of children in Taiwan had nasal MRSA colonization, we conducted this study to better understand the extent of nasal MRSA carriage rate among pediatricians in Taiwan who cared for these children.

## Materials and Methods

The study was approved by the institutional review board of Chang Gung Memorial Hospital. All participants of the Joint Meeting of 196th Annual Meeting of Taiwan Pediatric Association and 6^th^ Annual Conference of Asian Society of Pediatric Research, April 23-26, 2010, which were held in Taipei, Taiwan, were eligible and were invited to participate in this study. A total of 220 pediatricians from Taiwan were recruited and sampled from the nares after a written consent was obtained and a questionnaire including the demographics and medical facilities they work for was obtained. 

For each subject, nasal swab specimen was collected from the anterior nares using 2 separate dry Copan Transystem Liquid Stuart swabs (Venturi Transystem; Copan Diagnostics, Corona, CA). Each swab was rubbed inside the anterior nares, first into one side and then into the other, ensuring that each swab contained specimens of both nares of each subject. These swabs were then transported at room temperature and processed within 4 hours. A polymerase chain reaction test (BD GeneOhm^TM^ Staph SR Assay; Becton Dickinson, NJ, USA) was used to detect MRSA first. For those with positive PCR results, the swabs were further put into Mueller Hinton Broth (Becton, Dickinson and Co.) in CO_2_ incubator at 37°C overnight and then was subcultured into TSA II 5% SB plate (Becton, Dickinson and Comapany, Sparks, MD) to obtain MRSA isolates for molecular characterization. After incubation and subcultivation, coagulase test were conducted by using rabbit plasma to ensure the identification of *S. aureus*. Cefoxitin test was then used to distinguish the MRSA from methicillin-susceptible *S. aureus* (MSSA) based on the recommendation of Clinical and Laboratory Standards Institute (CLSI) [10]. Once MRSA was isolated, the strains were frozen until the processing for molecular characterization.

 The susceptibility test was performed on Mueller–Hinton agar with disk-diffusion method following the protocol of CLSI [10] and included oxacillin, doxycyclin, vancomycin, teicoplanin, penicillin, trimethoprim/sulfamethoxazole, erythromycin, clindamycin, linezolid, fusidic acid [10]. 

The extraction and purification of MRSA chromosomal DNA were conducted by using DNA kit (QIAamp^®^ DNA Blood Mini Kit, QIAGEN). Pulsed-field gel electrophoresis (PFGE) with *Sma*I digestion was used to fingerprint all MRSA isolates according to the procedure described previously [11,12]. Staphylococcal chromosome cassette *mec* (SCC*mec*) type, and the presence of Panton-Valentine leukocidin (PVL) genes were determined by PCR assays according to the procedure described previously [11-15]. Multilocus sequence typing (MLST) [16] and *spa* typing [17] were performed for strains of representative PFGE patterns as described elsewhere. The procedure for the detection of *sasX* gene followed that described by Li et al and the primers used were *sasX*-f agaattagaagtacgtctaaatgc and *sasX*-r gctgattatgtaaatgactcaaatg [18].

 Statistical analyses were performed with the Statistical Package for the Social Sciences (SPSS software for Windows, version 15.0). Statistical significance was based on a significance level of less than 0.05.

## Results

Of the 220 participants, 139 (63.2%) were male. Most attendees aged between 31-60 years (80%). Table 1 illustrates the demographics and medical facilities of the participants. 66 participants (30%) worked in medical centers, and 94 (42.7%) worked in clinics. 

**Table 1:** Comparison of demographics between participants with and without methicillin-resistant *Staphylococcus aureus* colonization.

Characteristics	Total (n=220) No. (%)	Colonized (n=15) No. (%)	Un-colonized (n=205) No. (%)	*p* value
Male gender	139 (63.2)	11 (73)	128 (62)	NS
Age (yrs)				
<30	8 (3.6)	0	8 (3.9)	NS
31-40	78 (35.5)	7 (47)	71 (35)	NS
41-50	55 (25.0)	2 (13)	53 (26)	NS
51-60	42 (19.1)	2 (13)	40 (20)	NS
>60	37 (16.8)	4 (27)	33 (16)	NS
Medical career (yrs)				
<5	14 (6.4)	2 (13)	12(5.9)	NS
6-10	56 (25.5)	4 (26)	52 (25)	NS
11-20	60 (27.3)	4 (26)	56 (27)	NS
21-30	46 (20.9)	3 (20)	43 (21)	NS
>30	44 (20.0)	2 (13)	42 (20)	NS
Clinician	210 (95.5)	14 (93)	196 (96)	NS
Health care units				
Medical center	66 (30.0)	1 (6.7)	65 (32)	0.041
Regional hospital	35 (15.9)	5 (33)	30 (15)	0.056
Local hospital	19 (8.6)	1 (6.7)	18 (8.8)	NS
Clinics	94 (42.7)	8 (53)	86 (42)	NS
Others	6 (2.7)	0	6 (2.9)	NS

MRSA was detected by PCR in 15 participants, with a carriage rate of 6.8%. Comparing the demographics between those with and without MRSA colonization (Table 1), we found that MRSA-colonized participants had a significantly lower rate of working in the medical center (p=0.041), while a higher rate of working in the regional hospital (p=0.056), than those without MRSA colonization. 

**Figure 1:**
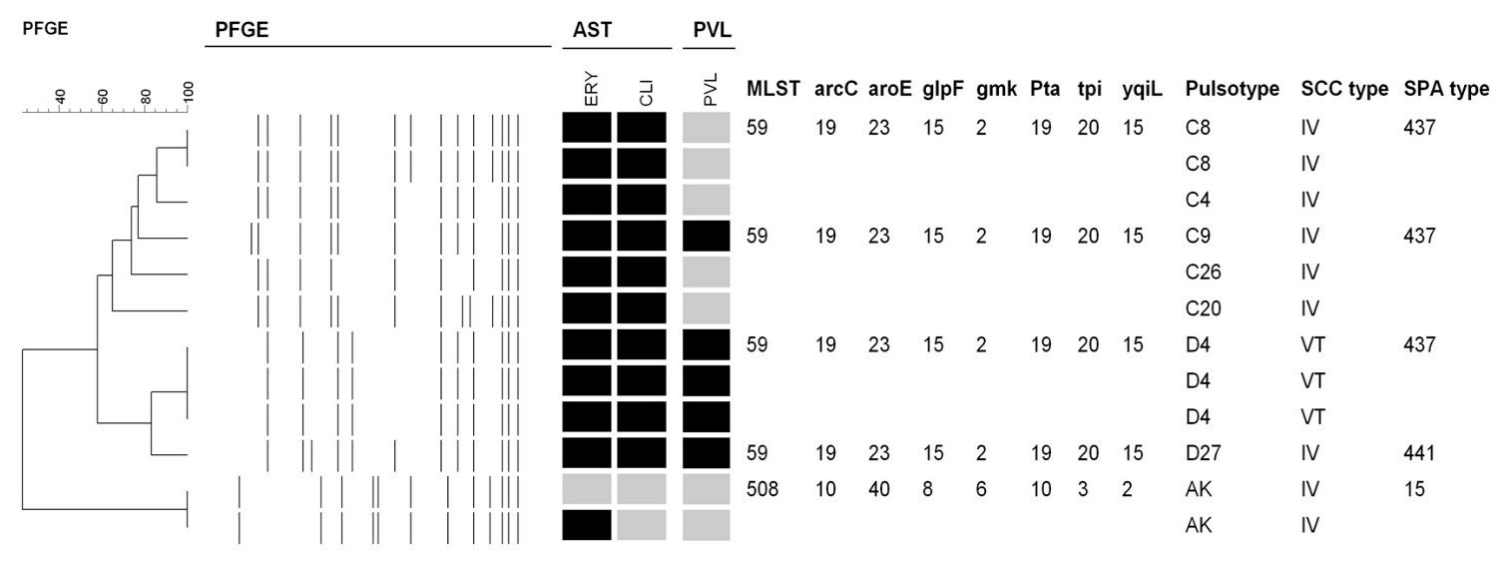
Molecular characterization of the 12 MRSA isolates. All 12 isolates were susceptible to vancomycin, teicoplanin, linezolid, fusidic acid, trimethoprim/sulfamethoxazole, and doxycyclin, and resistant to penicillin. Antimicrobial susceptibility tests (AST): black indicates resistance, and grey indicates susceptibility. Abbreviations are as follows: clindamycin (CLI), and erythromycin (ERY). PFGE, pulsed-field gel electrophoresis. PVL: black indicates that the Panton-Valentine leukocidin genes were detected. SCC*mec*, staphylococcal cassette chromosome mec elements; MLST, multilocus sequence type.

The nasal specimens from the PCR-positive participants were further cultured and MRSA were isolated from 12 specimens. All 12 isolates were molecularly characterized and the detailed molecular characteristics and antibiograms of the 12 MRSA isolates are shown in Figure 1. Three PFGE patterns (type C for 6 isolates, type D for 4 and type AK for 2), two sequence types (ST 59 for PFGE type C and D, and ST 508 for PFGE type AK), three spa types (t437 and t441 for ST 59, and t15 for ST 508/PFGE type AK), and two SCC*mec* types (IV for 9 isolates and V_T_ for 3) were identified, respectively. Three isolates carrying SCC*mec* V_T_ and two isolates with SCC*mec* IV were PVL-positive. All 12 isolates were susceptible to vancomycin, teicoplanin, trimethoprim/sulfamethoxazole, linezolid, fusidic acid, and doxycyclin, and resistant to penicillin, respectively (Figure 1). Only two isolates were susceptible to clindamycin and one of them was also susceptible to erythromycin. None of the 12 MRSA isolates carried the *sasX* gene.

## Discussion

Results from the present study indicate that 6.8% of pediatricians in Taiwan had MRSA carriage in the nares. The rate was comparable to that among HCWs reported from Taiwan previously, which ranged from 5.0% among those working in ordinary wards to 7.8% among those working in endemic neonatal intensive care units [19-21]. However, the rate of nasal MRSA carriage for pediatricians in the present study was higher than that for general adult population in Taiwan. For example, the rate of nasal MRSA carriage was 3.6% for healthy adults [21], 3.8% for 296 adult patients receiving hemodialysis [22], 3.8% for 502 adult patients visiting emergency room [23], 3.8% for 3098 adults for health examination [24] and 4.8% for 500 parturient mothers [25]. But the rate was lower than that for healthy children, ranging from 6.2% to 9.5% between 2005 and 2008 [26]. But, for contacts, previous reports from Taiwan indicated that the nasal MRSA carriage rate was found to be 13.6% for 66 contacts with a severe case of CA-MRSA infection [20], and 25%for 121 household contacts [27]. The issue whether contact with children or patients with MRSA infection is associated with a higher MRSA carriage rate for pediatricians needs further investigations.

In the present study, we used the BD GeneOhm StaphSR assay to detect nasal MRSA carriage first. As a screening method, the BD GeneOhm StaphSR assay could rapidly detect MRSA colonization in humans. In our previous study [28], we found a negative result of the assay could almost exclude *S. aureus* colonization, while a positive result should require culture to confirm it. However, Bartels et al [29] reported that some MRSA isolates with specific SCCmec could be missed by the BD GeneOhm StaphSR assay. 

All the MRSA isolates available for molecular characterization were found to carry either type IV or V SCC*mec*, which suggests the isolates are community strains. Most isolates, though not as multi-resistant as HA-MRSA, were resistant to clindamycin and erythromycin. Further characterizations demonstrated that 10 of the 12 isolates belonged to ST 59 which is a typical community strains in Taiwan [8,13,26]. Again, these findings partly reflected that CA-MRSA was prevalent in Taiwan.

*Sas*X gene is a novel staphylococcal gene encoding a surface-anchored protein and was increasingly identified in the MRSA strains of ST239 background during 2003 and 2011 in China [18]. It has been demonstrated that the *Sas*X protein can promote nasal colonization and enhance virulence of *S. aureus* and was further proposed as the crucial factor contributing to the MRSA epidemic in Asia [18,30]. None of the 12 MRSA isolates in the present study carried this gene, which indicated that there was no evidence of transfer of *sasX* gene from ST239 MRSA isolates to those with other genetic background yet.

The role of HCWs on MRSA transmission is the source, vector or victim, which is an issue needed to be elucidated and explored. In addition, it is intriguing that HCWs working in medical centers had a significant lower nasal MRSA carriage rate, while those working in regional hospitals had a borderline significant higher carriage rate. This finding as well as its clinical implication needs to be further delineated.
